# Single-center real-world data and technical considerations from 100 consecutive patients treated with the Perceval aortic bioprosthesis

**DOI:** 10.3389/fcvm.2024.1417617

**Published:** 2024-07-12

**Authors:** Hannes Müller, Philipp Szalkiewicz, Peter Benedikt, Thomas Ratschiller, Bruno Schachner, Sophie Schröckenstein, Andreas Zierer

**Affiliations:** Department of Cardio-Vascular and Thoracic Surgery, Kepler University Hospital—Faculty of Medicine, Johannes Kepler University, Linz, Austria

**Keywords:** aortic valve replacement, Perceval aortic valve, aortic valve surgery, biologic aortic valve, sutureless aortic valve prosthesis

## Abstract

**Objectives:**

Although the Perceval sutureless aortic valve bioprosthesis presents a feasible alternative to conventional aortic valve prostheses, the extent of its applicability with respect to technical considerations for a real-world patient collective is still under debate.

**Methods:**

One hundred patients received the Perceval prosthesis [males: 59; age: 72.5 (7.3–79) years] between December 2015 and February 2023 [EuroSCORE II: 2.8 (1.7–5.4)] for an aortic valve replacement (AVR), with additional concomitant procedures, for underlying severe aortic valve stenosis [*n* = 93 (93)], endocarditis [*n* = 5 (5)], and redo AVR [*n* = 7 (7)] including a prior surgical AVR [*n *=* *4 (4)] and a failed transcatheter aortic valve implantation [*n *=* *3 (3)]. Surgery was conducted primarily by median sternotomy [*n *=* *71 (71)] and, alternatively, by the upper hemisternotomy approach [*n* = 29 (29)].

**Results:**

Over a median follow-up time of 36.5 (16.5–53) months, eight patients (8%) underwent postoperative pacemaker implantation, with five (5%) due to high-grade atrioventricular block, while nine patients experienced a stroke (9%). The median values of maximum and mean gradients across all valve sizes were 22 (18–27.5) mmHg and 10 (13–18) mmHg, respectively. Two patients (2%) had moderate and one (1%) had severe paravalvular leakage, with the latter presenting the only case of underlying valve migration and induced redo AVR with valve explantation 2 days following initial surgery. Thirty-day mortality (and overall mortality) was 5% and 26%, respectively.

**Conclusion:**

The implantation of the Perceval bioprosthesis is feasible for a variety of indications, with excellent hemodynamic results and low complication rates in a real-world high-risk patient collective.

## Introduction

Although the Perceval aortic valve bioprosthesis is widely applied as a sutureless aortic valve (SAV) prosthesis for patients undergoing surgical aortic valve replacement (AVR), published data on its real-world application are currently scarce.

AVR is considered a standard procedure in cardiac surgery; however, it is also associated with increased complications in a substantial number of high-risk patients deemed as non-ideal candidates for transcatheter aortic valve implantation (TAVI) in clinical practice.

By introducing rapid deployment valves (RDV), perioperative cardiac ischemia times have been successfully reduced to those seen with conventional aortic valve prosthesis (cAVP) (1), an established risk factor for adverse outcomes (2–4), making it particularly attractive in minimally invasive access approaches and for the high-risk patient collective (3).

Despite including the Perceval bioprosthesis in large multicenter trials (1), which demonstrate its overall feasibility and comparative viability to traditional sutured aortic valves with promising long-term results (5), data and technical considerations for more explicit use in patients experiencing versatile indications, or those undergoing multivalvular or redo procedures, are underreported in the current literature and are primarily limited to case reports (6, 7).

Amid increasingly elaborate patient evaluation in an era when alternative procedures for AVR are emerging, the range of applicability of RDV is a topic of an ongoing debate.

This study provides a real-world analysis and technical considerations regarding patients undergoing aortic valve replacement using the Perceval bioprosthesis across a broad range of indications and concomitant procedures.

## Methods

### Study design

This study is a retrospective, single-center analysis covering all patients who received aortic valve replacements at the Department of Cardio-Vascular and Thoracic Surgery at Kepler University Hospital—Faculty of Medicine, Johannes Kepler University, Austria, between December 2015 and December 2023, using the Perceval Sutureless Heart Valve® (Corcym UK Limited, London, UK) [Perceval S: *n* = 98 (98%)] and the recently introduced new-generation Perceval PLUS Sutureless Heat Valve® (Corcym UK Limited, London, UK) [Perceval Plus: *n* = 2 (2%)]. The postprocedural follow-up was conducted on an outpatient basis. In addition, mortality data have been documented with the department´s annual data request from the mortality registry of Statistic Austria (STAT)—the Austrian statistical office. The study protocol was approved by the local ethics committee.

### Definition of outcome parameters

A stroke was defined as any postoperative sensomotoric deficit showing cerebral correlates detected by computed tomography or magnetic resonance imaging. A transient ischemic attack (TIA) was defined as similar symptoms without any detectable cerebral correlates on the given imaging modalities used. The corresponding evaluations were conducted by a neurologist and radiologist.

### Perceval aortic valves

The Perceval S and Perceval Plus aortic valve bioprostheses are made from bovine pericardium and designed for sutureless replacement of the aortic valve. The nitinol stent alloy is elastic and retains its shape, facilitating prosthesis placement using a self-expanding and self-anchoring valve stent at the aortic root and the sinus of Valsalva. The carbofilm coating inhibits inflammatory reactions while simultaneously promoting stent frame endothelialization. The upper and lower crown-shaped ring segments are used to position the valve in the aortic annulus and sinotubular junction (STJ), with the sinusoidal struts promoting stabilization within the aortic sinuses. Three inner columns connect the individual ring segments to which the commissures of the pericardial valve leaflets are attached. The recently introduced new-generation Perceval Plus incorporates an improved tissue treatment and a compact design with reduced prosthesis height; this design intends to reduce valve degeneration and valve ventricular protrusion. The prosthesis is available in four sizes: S, 19–21 mm; M, 22–23 mm; L, 24–25 mm; XL, 27 mm.

### Surgical considerations

The involved center prefers aortic valve replacement using the Perceval aortic valve bioprosthesis to shorten perioperative myocardial ischemia time in patients considered to be high-risk and frail on account of underlying comorbidities and who are likely to experience unfavorable surgical outcomes as a result. This includes patients requiring concomitant cardiac procedures or redo aortic valve replacement and hostile aortic roots, such as selective application in aortic annuli of small diameter, severe calcification, and previous stentless aortic valve replacement, including any previous aortic homograft repair.

### Surgical techniques and valve placement

**Figure 1:**
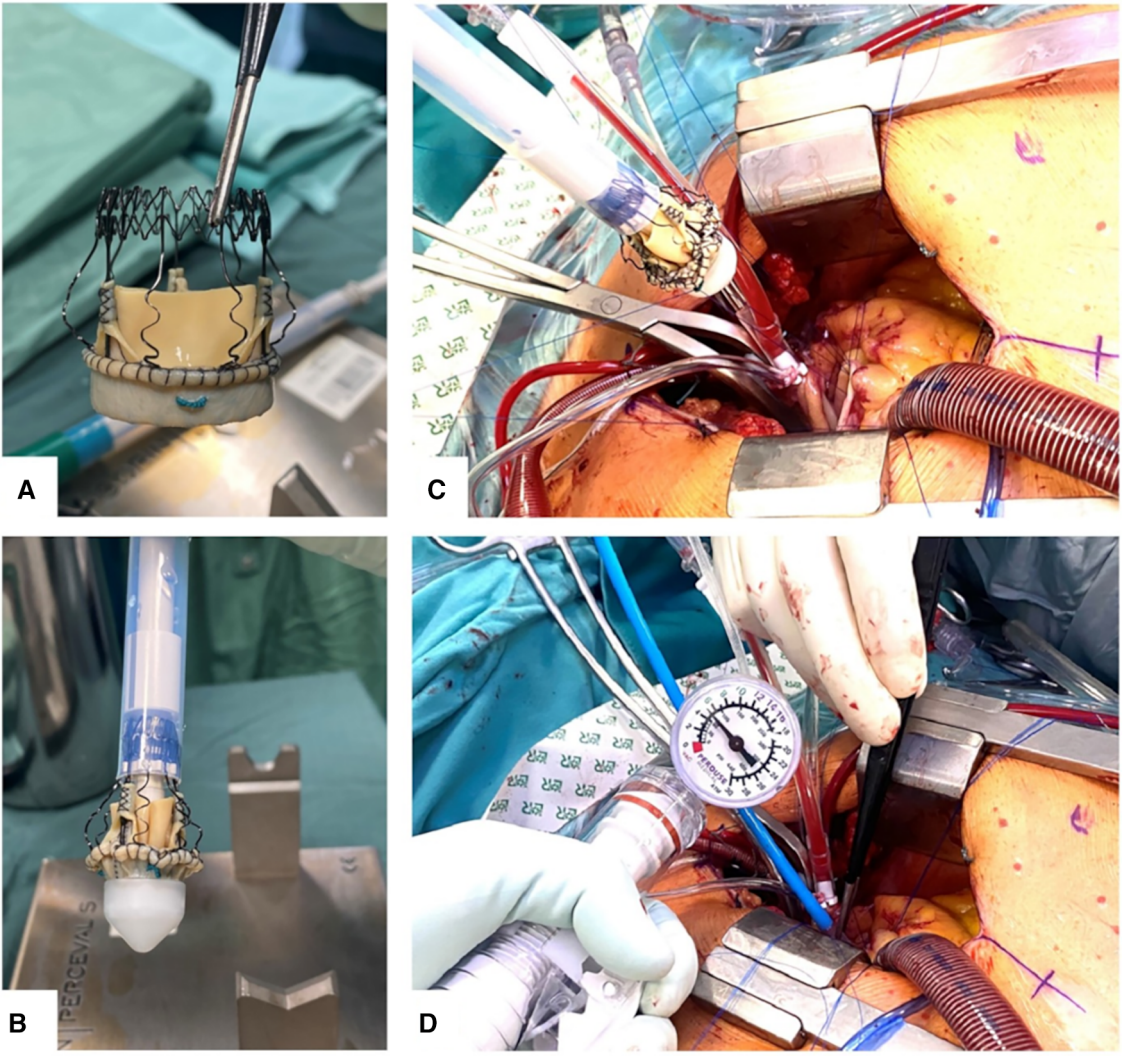
Perceval S aortic valve bioprosthesis in the expanded state (**A**) and crimped state loaded onto the specific holder accessory (**B**). Implantation of Perceval S by parachuting on placed guiding sutures during the hemisternotomy approach (**C**) and inflation of its valvuloplasty balloon by required pressure applied (**D**).

The surgical incision involved traditional surgical approaches. During median sternotomy, the sternum was incised and retracted at the midline, and during hemisternotomy, the incision was laterally deflected to the left side at the fourth intercostal space. Arterial cannulation was performed in the ascending aorta, aortic arch, or directly in the subclavian artery, depending on aortic calcium distribution. Venous drainage was conducted by right atrial cannulation. Following aortic cross-sectional clamping at the distal ascending aorta and either antegrade or concomitant retrograde St. Thomas cardioplegia, a transverse aortic incision was made distal to the STJ, 3.5 cm from the aortic annulus. The native aortic valve leaflets were excised, and the aortic root was decalcified. The aortic valve was then collapsed onto its specific support accessory and inserted into the aortic annulus by way of three appropriately placed guiding sutures, of which one was placed at each aortic sinus along the resection line of the native aortic valve and passed through the valve’s customized thread loops. After positioning the inflow ring of the aortic valve at the designated landing point, the prosthesis was released, allowing for continuous self-expansion at the aortic annulus and subsequent postdilatation using its specific postdilatation catheter. Balloon inflation was carried out using 4 units of atmospheric pressure applied for 30 s (Figure 1). The respective guiding sutures were then removed. The placement protocol was standardized and applied to every patient for respective analysis, without any additional procedural modifications.

### Statistical analysis

The categorical data are displayed as numbers and corresponding percentages, while continuous variables appear as mean and standard deviation (SD) or median and interquartile range (IQR), depending on their distribution. Statistical analysis was conducted using SPSS statistical software, version 26 (IBM Corp., Armonk, NY, USA).

## Results

### Patient baseline characteristics

Of the 100 patients who received the Perceval aortic valve prosthesis [Perceval S: *n* = 98 (98%); Perceval Plus: *n* = 2; (2%)], 59 (59%) were men, with a median age of 72.5 (67.3–79) years and a median EuroSCORE II of 2.8 (1.7–5.4); notably, EuroSCORE II values of ≥4.0% were observed in 35 (35%) patients. The underlying surgical indications for aortic valve replacement were aortic valve stenosis in 93 (93%), aortic insufficiency in 37 (37%), and endocarditis in 5 (5%) patients, including 1 (1%) with prosthetic aortic valve endocarditis. Seven patients (7%) had undergone prior cardiac surgery, with two patients (2%) undergoing coronary artery bypass grafting (CABG), one (1%) undergoing ventricular septal defect repair, one (1%) undergoing aortic homograft repair, and three (3%) undergoing surgical prosthetic aortic valve replacement. Moreover, three patients (3%) had undergone immediate acute Perceval implantation following prior transcatheter aortic valve implantation (TAVI) for severe paravalvular leakage (PVL), two patients one month each and one patient two years after the interventional valve replacement, respectively. Seven patients (7%) had undergone prior aortic valve replacement surgery or intervention (Table 1).

**Table 1:** Patient baseline characteristics.

*n* = (all, %)	100 (100)
Patient’s risk profile	
Male	59 (59)
Age, median (IQR)	72.5 (67.3–79)
BMI, median (IQR)	27.1 (24.7–30.3)
EuroSCORE II, median (IQR)	2.8 (1.7–5.4)
EuroSCORE II ≥4%	35 (35)
Underlying patient conditions	
COPD	10 (10
Creatinine (mg/dl), median (IQR)	1 (0.9–1.2)
GFR (ml/min/1.73 m^2^), median (IQR)	71.2 (56.3–79.4)
Preserved left ventricular ejection fraction	73 (73)
Hyperlipidemia	47 (47)
Hypertension	61 (61)
Diabetes mellitus type II	24 (24)
Insulin-dependent diabetes mellitus type II	4 (4)
Peripheral arterial disease	15 (15)
Carotid artery disease	17 (17)
Coronary heart disease	58 (58)
Myocardial infarction	10 (10)
Prior stroke	4 (4)
Atrial fibrillation	20 (20)
Endocarditis	5 (5)
Pulmonary embolism	2 (2)
Jehovah's witness	5 (5)
Aortic valve stenosis	93 (93)
Mitral valve stenosis	0
Aortic valve insufficiency	37 (37)
Mitral valve insufficiency	38 (38)
Prior cardiac procedures	7 (7)
Overall aortic valve procedures	7 (7)
Prior surgical aortic valve replacement	4 (4)
Prior TAVI	3 (3)
Prior CABG	2 (2)
Prior aortic root homograft	1 (1)
Prior VSD repair	1 (1)
Prior PMI	7 (7)

CABG, coronary artery bypass grafting; COPD, chronic obstructive pulmonary disease; EuroSCORE II, updated European system for cardiac operative risk evaluation; GFR, glomerular filtration rate; IQR, interquartile range; PMI, pacemaker implantation; TAVI, transcatheter aortic valve implantation; VSD, ventricular septal defect.

### Procedural characteristics

Eighty-five patients (85%) underwent elective Perceval implantation, while 49 (49%) received simultaneous cardiac procedures, including CABG in 47 (47%), mitral valve replacement in 3 (3%), tricuspid valve replacement in 1 (1%), and ascending aortic replacement in 1 (1%) patient. The majority of patients underwent surgery by a standardized median sternotomy approach [*n* = 71 (71%)], while the upper hemisternotomy approach was used in 29 (29%) patients. In the overwhelming majority of patients [*n* = 94 (94%)], arterial cannulation was performed on the ascending aorta, while aortic arch and right subclavian artery cannulation was performed in two (2%) and four (4%) patients, respectively. The overall median cardiopulmonary bypass (CPB) time was 99 (78–137) min, and the median aortic cross-clamping (ACC) time was about 42.5 (34–59) min (Table 2).

**Table 2:** Procedural characteristics.

*n* = (all, %)	100 (100)
Elective	85 (85)
Concomitant cardiac procedures	49 (49)
Mitral valve surgery	3 (3)
Tricuspid valve surgery	1 (1)
Coronary artery bypass grafting	47 (47)
Ascending aortic replacement	1 (1)
Carotid endarterectomy	6 (6)
Access site	
Median sternotomy	71 (71)
Upper hemisternotomy	29 (29)
Cannulation	
Ascending aorta	94 (94)
Aortic arch	2 (2)
Right subclavian artery	4 (4)
Cardiac ischemia times	
CPB (min), median (IQR)	99 (78–137)
ACC (min), median (IQR)	42.5 (34–59)

ACC, aortic cross-clamp time; CPB, cardiopulmonary bypass time.

### Outcomes

**Figure 2:**
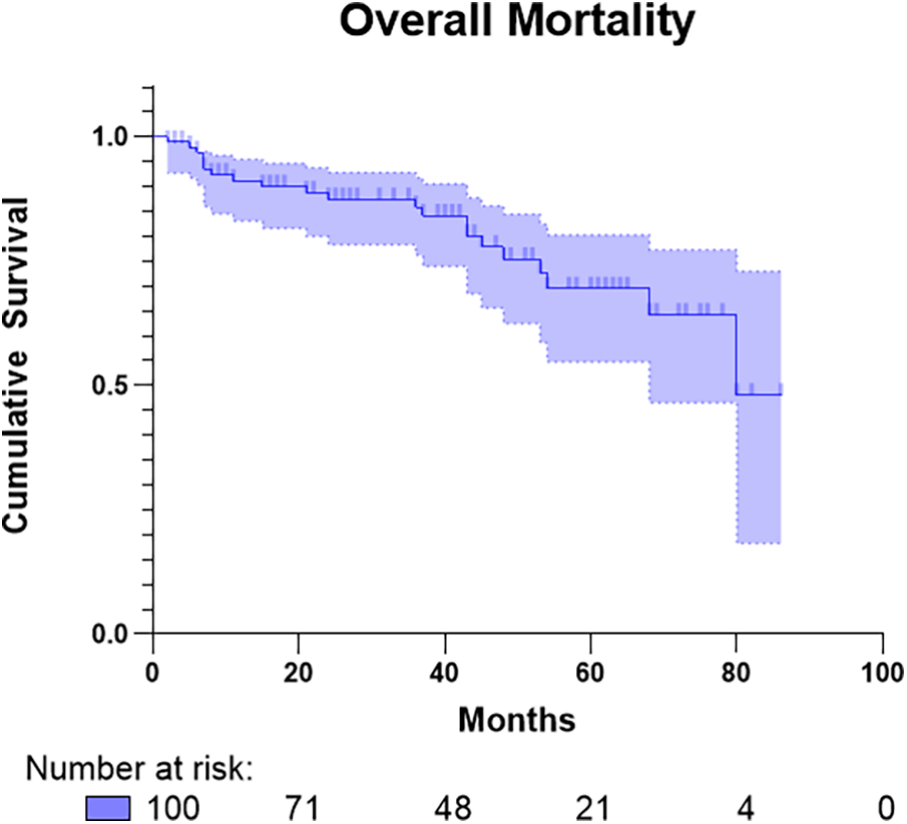
Kaplan–Meier graph of overall cumulative survival across follow-up period in months.

The average length of stay in the intensive care unit (ICU) and the hospital was 3 (2–5) and 16 (12–22) days, respectively. Among all the patients, one (1%) had a repeat aortic valve surgery with a stented aortic valve bioprosthesis 2 days after undergoing the initial Perceval implantation. Shortly after surgery, the patient was hemodynamically unstable, showing severe PVL on the right coronary cusp, which was detected by transesophageal echocardiography and caused by upward valve migration of 3 mm. The Perceval prosthesis was successfully explanted during emergency surgery without complications. In terms of early hemodynamic outcomes, the current patient represented the only case of severe PVL observed before discharge from the hospital; additionally, one out of two patients (2%) underwent a cardiac procedure after initial aortic valve surgery, with one patient (1%) undergoing mitral valve replacement using a Tendyne® mitral valve prosthesis (Abbott Park, North Chicago, IL, USA) after 18 months. Two additional patients (2%) had moderate PVL, and two (2%) had mild PVL. The median values of maximum and median aortic valve gradients prior to hospital discharge were reported as 22 (18–27.5) mmHg and 10 (13–18) mmHg, respectively. An overview of the number and postoperative aortic valve gradients for each implanted valve size is provided in Table 3. Five patients (5%) experienced pericardial tamponade, of which three (3%) underwent surgical pericardial revision, while five patients (5%) underwent hemothorax revision by resternotomy postsurgery. In terms of conduction disturbances, five patients (5%) had third-degree atrioventricular (AV) and eight patients (8%) underwent pacemaker implantation (PMI) during follow-up. Nine patients (9%) experienced postoperative stroke, three patients (3%) received postoperative mechanical circulatory support by extracorporeal membrane oxygenation (ECMO), and one patient (1%) needed concomitant intra-aortic balloon pump application. Over a median follow-up period of 36.5 (16.5–53) months, 26 (26%) patients died (Figure 2), with a median time-to-event duration of 13 (5–43) months. It is noteworthy that these patients underwent additional concomitant cardiac procedures during the initial surgery [*n* = 17 (65.4%) vs. *n *=* *32 (43.2%); *p *=* *0.052]. Among them, five (5%) experienced early mortality within 30 days after the initial surgery, all prior to hospital discharge. Four patients (4%) died due to hemodynamic failure, with two (2%) on ECMO support and underlying acute respiratory distress syndrome each and one patient (1%) each due to perioperative cerebral multiembolic events and aspiration pneumonia, respectively (Table 4).

**Table 3:** Implanted Perceval valve size and according postoperative aortic valve gradients.

Valve size	*n* = 100 (%)	Maximum gradient (mmHg), median (IQR)	Mean gradient (mmHg), median (IQR)
S	8 (8)	25.5 (19.8–30.5)	14.5 (10.8–20.5)
M	27 (27)	27.5 (20.5–31)	18 (12–20)
L	31 (31)	22 (22–25)	13 (10–15)
XL	34 (34)	20 (16–24.3)	12.5 (9.3–15.8)

IQR, interquartile range.

**Table 4:** Postoperative adverse events.

*n* = (all, %)	100 (100)
Redo aortic valve replacement	1 (1)
Redo cardiac surgery	2 (2)
Valve migration	1 (1)
Myocardial infarction	0 (0)
Pericardial tamponade	5 (5)
Pericardial revision	3 (3)
Hemothorax revision	5 (5)
Third-degree AV block	5 (5)
Asystole	4 (4)
TIA	3 (3)
Stroke	9 (9)
Stroke <30 days	7 (7)
PMI	8 (8)
Dialysis	4 (4)
ECMO	3 (3)
Mortality overall	26 (26)
Mortality within 30 days	5 (5)
Hemodynamic outcomes	
Maximum gradient (mmHg), median (IQR)	22 (18–27.5)
Mean gradient (mmHg), median (IQR)	10 (13–18)
PVL	
Trace	2 (2)
Moderate	2 (2)
Severe	1 (1)

ECMO, extracorporeal membrane oxygenation; IQR, interquartile range; PMI, pacemaker implantation; PVL, paravalvular leakage; TIA, transient ischemic attack.

## Discussion

Based on this study, AVR using the Perceval bioprosthetic aortic valve delivers excellent results with adequate postoperative valve hemodynamics across a wide range of indications and concomitant procedures.

Based on its underlying caged stent frame design incorporating nitinol-based shape memory, its feasibility is particularly favored for high-risk patients, as well as in cases of small, hostile, and highly calcified aortic roots. While its unique prosthetic design featuring a collapsed valve facilitates excellent visualization and deployment control during implantation and is well-suited for complex anatomical characteristics of the aortic root and minimally invasive access site approaches, its use needs to be weighed against the potential risk of PMI and valve migration, with regard to anatomic aortic root properties.

As implantation techniques rely on an expandable valve frame similar to those used in TAVI prostheses, preoperative aortic root anatomy assessment is critical because the nitinol alloy in self-expanding TAVI valves could potentially result in damage to the surrounding tissue (8), which, in correlation with calcium distribution (9), anatomical landmarks, (10) and preoperative conduction disturbances (11), may potentially lead to significantly higher PMI rates in RDV (approximately 9.1% in large multicenter registries) (1), which correspond with the reported 8% in the present analysis. Park et al. revealed that RDV oversizing with consecutive PVL and high-grade atrioventricular block when using the Edwards INTUITY Elite valve system® (Edwards Lifesciences, Irvine, CA, USA) is inversely correlated to aortic root and left ventricular outflow tract (LVOT) diameters and concluded that patients with eccentric aortic roots should be considered at increased risk for underlying inadequate prosthesis sizing (12).

In addition, ensuring the integrity of both anchoring sites, with respect to a required diameter ratio between the two that does not exceed 1 to 1.3, is essential; otherwise, there is an increased risk of prosthesis malexpansion and PVL, as well as inadequate landing zone anchoring and a subsequent risk of prosthesis migration. We therefore performed the aortotomy at a distance of 3.5 cm from the aortic annulus, as recommended, further distally compared to cAVP implantation. Despite aortic aneurysms being consequently regarded as a contraindication, we are reporting on the feasibility of concomitant supracoronary ascending aortic aneurysm repair in one patient.

In this regard, relatively small aortomitral continuity has been considered a risk factor for the suboptimal placement of guiding sutures due to underlying interference with its commissural struts, which might cause impinging on the left ventricle in given anatomic properties, as reported during TAVI (13), with supra-annular valve placement in the presence of a bioprosthetic mitral valve and consequent consecutive prosthesis migration (6). Nevertheless, in the current analysis, we report an overall satisfactory experience with Perceval prostheses implantation in patients treated with repaired or replaced mitral valves without requiring additional adaptations in our prespecified standardized procedural protocol.

However, we also report on one patient who experienced prosthesis migration and acute valve explant as a result of underlying severe PVL. As consideration was given to valve sizing with respect to diameters of given anchoring sites, we hypothesize an underlying valve pop-up as a consequence of prosthesis malexpansion after insufficient debridement of a severe annular calcification, as well as initial mispositioning of the prosthesis in complex anatomic aortic root properties with a relatively small aortomitral continuity.

On the other hand, the Perceval’s lack of any prosthesis skirt or sewing ring and gradual expansion of the nitinol frame account for beneficial transvalvular gradients due to the underlying increase of the effective orifice area (EOA) compared to cAVP, with improved left ventricular hypertrophy regression (14), thereby considerably reducing the risk of patient prosthesis mismatch (PPM), which, in conjunction with the reduced procedural cardiac ischemia times (1), could exponentiate the survival benefit, especially in a high-risk patient collective, as reported in minimally invasive access site approaches (2, 3). In reporting on adequate hemodynamics across all valve sizes, we also regard the Perceval, along with its prosthetic features, as contributing to a more favorable outcome in high-risk patients in the present analysis and potentially competing with TAVI in first aortic valve procedures. The importance of a prosthetic design in providing hemodynamic benefits through supra-annular leaflet mounting is of major interest among patients with small aortic annuli who are prone to PPM and consecutively impaired mortality outcomes, according to the TAVI-Small registry data (15, 16). This was emphasized in the SURTAVI-TRIAL, which revealed larger EOA and superior transvalvular gradients in the Medtronic CoreValve® (Medtronic, Dublin, Leinster, Ireland) prosthesis compared to cAVP, with the outcome for the given intermediate-risk patient population being considered non-inferior despite comparatively higher PVL and PMI rates at midterm follow-up (17). However, when comparing the Medtronic CoreValve and the ACURATE aortic valve system® (Boston Scientific, Marlborough, MA, USA) to the new Perceval, Muneretto et al. reported improved perioperative and long-term transvalvular gradients, lower rates of PMI and PVL, and reduced major adverse cardiac adverse events and cardiac-related mortality in the surgical cohort, with TAVI being an independent risk factor in this regard, particularly in an intermediate-risk patient collective (18). Notably, Santarpino et al. reported that reduced PVL in the Perceval due to annular debridement compared to the Edwards SAPIEN XT® transcatheter heart valve (Edwards Lifesciences, Irvine, CA, USA) was also a contributing factor for improved survival in a high-risk patient collective (19).

**Figure 3:**
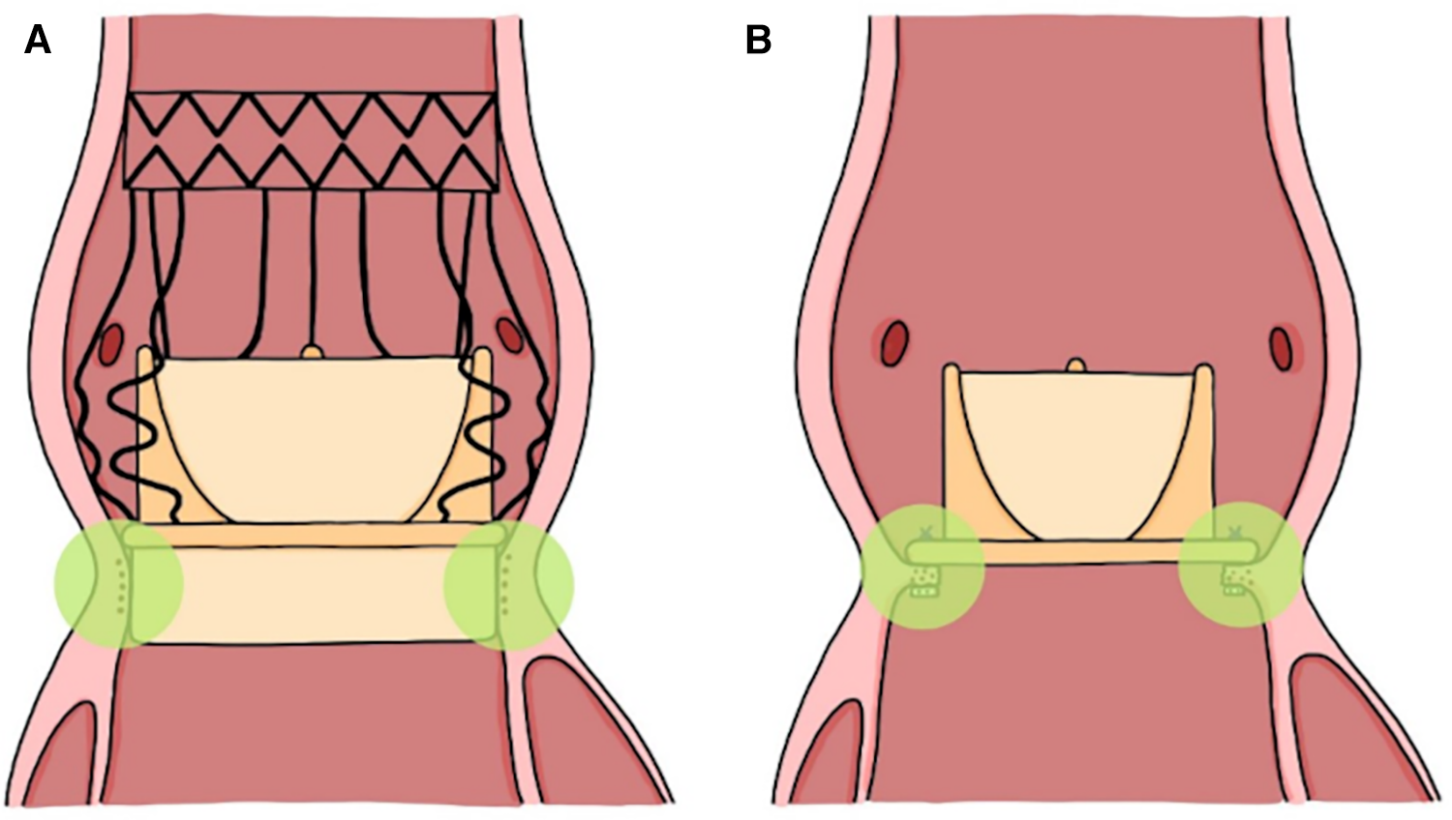
Implantation of the Perceval S aortic valve bioprosthesis along the resection line of the native aortic valve, covering thrombogenic hotspots (green area) at the aortic annulus (**A**) compared to the conventional aortic valve prosthesis (**B**).

Furthermore, PPM is a circumstance that is feared in patients admitted for redo valve-in-valve (VIV) procedures, particularly when associated with a previously small cAVP (20). Although redo AVR has been comparably advantageous in terms of lower rates of PPM and PVL (13, 21), the possible long-term outcomes have yet to be expected, as reported by Bleiziffer et al., who noted a poor estimated 8-year long-term survival rate of only 38% (22). In this regard, the Perceval caged stent frame design, featuring an expandable inflow ring, provides an ideal landing zone for TAVI prostheses, facilitating the implantation of larger TAVI valve sizes and preventing PPM with optimal gradients (23) while also potentially preventing coronary ostium obstruction, which, according to the VIVID registry, affects 2.3% of VIV procedures with an associated 30-day mortality rate of 52.9% (24). Its leaflets, mounted to the inner columns, limit leaflet displacement laterally, while the expansion force from its outer columns, with the absence of pledgeted sutures, potentially limits aortic root distortion with lowered annulo-ostial distance, as in the case with the use of pledgeted sutures in cAVP and Edwards INTUITY Elite implantation (25). Both of these are notable risk factors for coronary ostium obstruction, exacerbated in small aortic roots with short valve-to-coronary ostium distance (<4 mm) (24). Moreover, the self-expandable and pericardial-covered inflow ring covers the aortic valve resection line, a potential area of elevated thrombogenicity, potentially decreasing the risk of embolic incidents compared to cAVP, with its exposed aortic annulus containing possible residual calcification, pledgets, and sutures (Figure 3).

Given the limited amount of available data comparing redo AVR with RDV, the conclusive impact on the given patient population remains speculative. The RECORD registry regarding redo AVR has reported comparatively higher rates of intrahospital mortality of 5.1% and 4.5% for concomitant and isolated redo AVR to initial surgery, respectively, estimating CPB cutoffs of >165 min for adverse outcomes (26). According to the improved outcomes of minimally invasive techniques compared to standard surgical approaches (2, 3), Kaneko et al. demonstrated significant long-term mortality benefits in redo AVR conducted by the hemisternotomy approach compared to the median sternotomy approach (27). In this regard, we consider the advantages of the reduced CPB period and sutureless implantation technique of the Perceval as ideal for redo AVR, including in cases of failed TAVI, as a substantial number of patients are deemed unsuitable candidates for redo VIV procedures, which are associated with hazardous intrahospital mortality outcomes (28, 29). We report on three patients who underwent TAVI explantation for the Perceval valve, all due to underlying severe PVL. Aortotomy was conducted according to the given protocol with respect to 1–1.5 cm distal to the upper valve frame and explantation facilitated by deforming the given stent frame with a Kocher clamp, liberating the TAVI prosthesis from the annulus without shear damage. It is noteworthy that the given prosthesis explant in one patient did not require additional endarterectomy 2 years after the initial TAVI due to underlying neo-endothelialization, as reported by Fukuhara et al. (29). As we prefer the sutureless implantation technique of the Perceval prosthesis in given sensitive tissue, avoiding any additional pledgeted sutures, the underlying benefit favors placement in hostile aortic roots with high-grade calcification, such as in aortic homografts (7) or fragile aortic tissue (in conditions like endocarditis with paravalvular abscess) (30), while the absence of additional foreign material could reduce infectious areas that might lead to new or recurrent endocarditis.

We consider the relatively high stroke rates and intrahospital and overall mortality of 5% and 26%, respectively, to be a result of potential plaque embolization from severely calcified aortic roots, on the one hand, and to be indicative of the real-world, high-risk patient collective of the current analysis, on the other hand. It is noteworthy that in our center, the Perceval prosthesis is not considered the primary choice for aortic valve replacement but is rather implanted in multimorbid patients and those with complex anatomic aortic root properties and severe calcification. In this context, we refer to the relatively high number of patients with EuroSCORE II ≥4% [*n* = 35 (35%)] and the higher rate of concomitant cardiac procedures in patients who experience long-term mortality. Consequently, more research is needed to compare TAVI use and redo procedures in a currently aging and increasingly high-risk population of cardiac surgical patients.

## Limitations

This retrospective analysis was conducted without a control group. Hemodynamic data were not uniformly obtained during long-term follow-up, while cardiac-related mortality was not analyzed, as the cause of death could not be evaluated in each patient retrospectively after discharge from the hospital. Data on bicuspid aortic valves were not documented, as bias resulting from the absence of genetic analyses differentiating between genetically inherited and calcification-related bicuspid aortic valves could not be excluded.

## Conclusion

Real-world data obtained from all-comer patients undergoing AVR using the Perceval prosthesis reveal excellent short and long-term outcomes in terms of preventing early mortality and morbidity and providing optimal valve performance.

## Data Availability

The raw data supporting the conclusions of this article will be made available by the authors without undue reservation.
